# Preterm infants have significantly longer telomeres than their term born counterparts

**DOI:** 10.1371/journal.pone.0180082

**Published:** 2017-06-28

**Authors:** Vimal Vasu, Kara J. Turner, Shermi George, John Greenall, Predrag Slijepcevic, Darren K. Griffin

**Affiliations:** 1 Department of Child Health, East Kent Hospitals University Foundation NHS Trust, William Harvey Hospital, Ashford, Kent, United Kingdom; 2 University of Kent, School of Biosciences, Giles Lane, Canterbury, Kent, United Kingdom; 3 Brunel University London, Department of Life Sciences, College of Health and Life Sciences, Uxbridge, Middlesex, United Kingdom; University of Newcastle, UNITED KINGDOM

## Abstract

There are well-established morbidities associated with preterm birth including respiratory, neurocognitive and developmental disorders. However several others have recently emerged that characterise an ‘aged’ phenotype in the preterm infant by term-equivalent age. These include hypertension, insulin resistance and altered body fat distribution. Evidence shows that these morbidities persist into adult life, posing a significant public health concern. In this study, we measured relative telomere length in leukocytes as an indicator of biological ageing in 25 preterm infants at term equivalent age. Comparing our measurements with those from 22 preterm infants sampled at birth and from 31 term-born infants, we tested the hypothesis that by term equivalent age, preterm infants have significantly shorter telomeres (thus suggesting that they are prematurely aged). Our results demonstrate that relative telomere length is highly variable in newborn infants and is significantly negatively correlated with gestational age and birth weight in preterm infants. Further, longitudinal assessment in preterm infants who had telomere length measurements available at both birth and term age (n = 5) suggests that telomere attrition rate is negatively correlated with increasing gestational age. Contrary to our initial hypothesis however, relative telomere length was significantly *shortest* in the term born control group compared to both preterm groups and longest in the preterm at birth group. In addition, telomere lengths were not significantly different between preterm infants sampled at birth and those sampled at term equivalent age. These results indicate that other, as yet undetermined, factors may influence telomere length in the preterm born infant and raise the intriguing hypothesis that as preterm gestation declines, telomere attrition rate increases.

## Introduction

Preterm birth, defined by the World Health Organisation as birth at less than 37 weeks completed gestation, is estimated to account for 14.9 million or 11.1% of all births worldwide. Although survival rates are improving, recent data suggests that preterm birth is a risk factor in half of all neonatal deaths and that 1 million deaths each year are a direct result of complications associated with preterm birth [1, 2]. Furthermore, immediate and long term morbidity rates in those that survive have remained high and largely correlate with the degree of prematurity [1, 3–6].

By term age, preterm babies display a phenotype that is different from that of the term born infant. Specifically, these babies have altered adipose tissue partitioning [7, 8], ectopic fat deposition as intrahepatocellular lipid [9], hypertension [10–12] and insulin resistance [13, 14]. Moreover, there is preliminary evidence indicating that many of these morbidities persist into early adult life and as such, may represent a significant public health issue [15–18]. As the morbidities described are associated with ageing in adults, the observed phenotype of the preterm infant may be indicative of premature ageing. Attenuation or early recognition of this phenotype would therefore be desirable to reduce morbidity and to appropriately manage long-term health in these individuals.

Telomeres are repetitive DNA sequences at the end of chromosomes that become shorter with each cell cycle [19, 20]. When telomeres have shortened beyond a critical length, a DNA damage response is initiated, committing the cell to apoptosis or senescence [21, 22]. It is widely believed that an accumulation of senescent cells within a population leads to a loss of tissue function and ultimately organismal ageing [23]. Indeed, available data indicate a close negative correlation between chronological age and telomere length [24–28]. Furthermore, evidence supports a link between shortened telomeres and age-related morbidities in adults, many of which characterise the preterm infant phenotype described above [29–33].

Despite recent academic focus on telomere biology in newborns [34–49], little is known about telomere length and regulation in preterm infants. In summary the evidence to date demonstrates a reduction in telomere length with advancing gestational age, particularly in those born at less than 32 weeks completed gestation [50, 51]. This effect appears to be specific to life *ex utero*, since age matched foetuses do not conform to this trend [52]. Furthermore, other physiological events during labour, such as status of membrane rupture has also been shown to be relevant to telomere length in preterm infants [50, 53].

The *ex utero* environment of the neonatal intensive care unit differs fundamentally from both the *in utero* environment and the postnatal environment experienced by the term infant. For example, exposure of preterm infants to elevated levels of oxygen may potentially lead to increased reactive oxygen species and induce DNA damage (to which the G-rich telomere sequence is particularly susceptible) [54, 55]. In addition, altered nutrition, sleep cycles and general routine care procedures may induce increased levels of stress. In adults, similar stresses are associated with telomere attrition [56–59]. Therefore these and other *ex utero* factors might act to modulate telomere length in the preterm infant, resulting in a phenotype reminiscent of premature ageing.

Therefore, the aim of this study was to conduct a prospective observational study to compare telomere lengths of preterm infants sampled at birth and at term equivalent age with that of term infants. Although a small number of studies have compared telomere lengths in preterm infants with that of those born at term, none have assessed telomere length in preterm infants at term equivalent age (i.e. 37–42 weeks). Given that the evidence available demonstrate a reduction in telomere length with advancing gestational maturity in preterm infants and that the *ex utero* environment may be relevant, we sought to test the hypothesis that by term equivalent age, telomere length is shortened in preterm infants in comparison to term born infants.

## Materials and methods

With institutional research ethics committee approval (10/H1109/51) and informed parental consent we conducted a prospective observational study over a four year period (June 2011 to June 2015). A total of 47 preterm infants with a birth gestational age of < 32 completed weeks gestation were recruited from the level 3 (regional) neonatal unit at the William Harvey Hospital in Ashford, Kent and the level 1 neonatal unit at the Queen Elizabeth the Queen Mother Hospital in Margate, Kent. We chose to include preterm infants born < 32 weeks completed gestation in light of previous data, which showed a rapid and significant decline in telomere length with advancing gestational age in infants born at < 32 weeks [51]. In addition, this gestational age cut off is frequently utilised in other studies of preterm birth and allows for recruitment of a reasonable sample size within a reasonable time period. Of the 47 preterm infants sampled, 22 were sampled within 48 hours following birth and 25 were sampled at term equivalent age. In addition, a total of 31 term born infants who required blood sampling in the first 48 hours following birth for reasons such as evaluation of neonatal jaundice or suspected sepsis were recruited from the postnatal wards at these hospitals as a pragmatic comparator cohort. Babies were excluded from the study if they had an antenatal or postnatal diagnosis of a severe congenital malformation or were unlikely to survive. Once recruited, several demographic factors were collected from each participant. These included infant gestational age at birth, infant weight at birth and maternal age in all three groups.

### Telomere length measurement

1ml of additional blood was collected by venepuncture in a paediatric lithium heparin bottle taken at the time of routine blood sampling in study recruits. In preterm infants, we aimed to collect a sample within 48 hours of life or at term equivalent age (between 37–42 weeks post menstrual age). Term born infants underwent blood sampling at the time of routine blood sampling within the first 48 hours of life. Each blood sample was assigned a 2 digit numerical code and frozen at -80^°^C. Samples were collected by a member of the research team who was blinded to the group allocation of the blood sample and transported to the School of Biosciences, University of Kent under dry ice. Samples were thawed and equilibrated to room temperature prior to genomic DNA extraction using a DNA isolation from mammalian blood kit (Roche). Isolated DNA was dissolved in Tris-EDTA pH8.0 and assessed for DNA concentration and purity using a Nanodrop spectrophotometer. All samples were stored at -20^°^C until telomere length analysis by a single observer, using multiplex quantitative real time polymerase chain reaction (qRT-PCR). Primer design for telomere and a single copy reference gene (haemoglobin B) amplification was as previously described by Cawthon *et al* 2009 [60]. Simultaneous amplification of the telomere and single copy gene was achieved in a total reaction volume of 25μl using SensiMix^TM^ SYBR No-ROX Kit (Bioline), 50nM each primer and 25ng of standard or unknown sample DNA. The reaction was carried out using a Rotor-gene Q 2 plex HRM platform under the following cycling conditions: 95^°^C for 10 mins, two cycles of 94^°^C for 15 secs and 49^°^C for 15 secs, 37 cycles 94^°^C for 15 secs, 62^°^C for 10 secs, 74^°^C for 15 secs, 84^°^C for 10 secs and 88^°^C for 10 secs. Each unknown and standard DNA sample was assayed in triplicate. Relative telomere length expressed as telomere to single copy gene ratio (T/S ratio) was calculated using a standard comparative method (delta delta Ct) [61] (Equation 1). Intra- and inter-assay variations were 1.05% and 0.41% respectively.

T/S=2^((SampletelCt−SamplescgCt)−referencetelCt−referencescgCT))

#### Equation 1. Comparative method for the calculation of T/S ratio

‘Sample telCt’ refers to the unknown sample telomere sequence amplification cycle threshold, ‘Sample scgCt’ refers to the unknown sample single copy reference gene amplification cycle threshold, ‘reference telCt’ refers to the reference DNA telomere amplification cycle threshold and ‘reference scgCt’ is the reference DNA single copy gene amplification cycle threshold.

### Statistical analysis

Data were tested for normality using the Shapiro-Wilk test and analysed using SPSS version 21 (IBM Corp. Released 2012. IBM SPSS Statistics for Windows, Version 21.0. Armonk, NY: IBM Corp). The primary outcome, T/S, ratio was normally distributed and therefore parametric methods (univariate ANOVA) were used for analysis. Cohen’s d was used to determine effect size (small, medium, or large) for mean T/S ratio between the groups.

## Results

**Fig 1:**
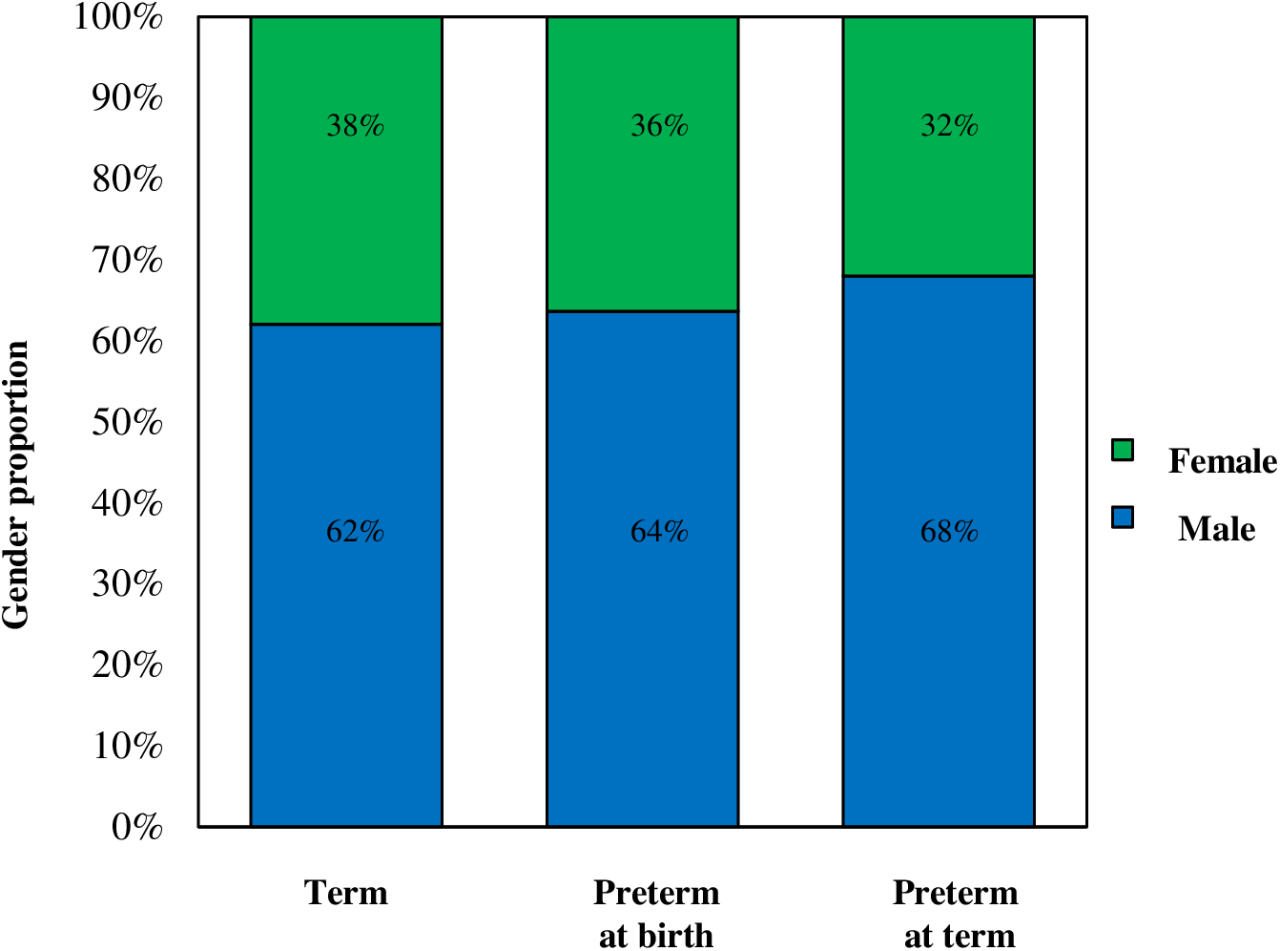
Proportion of males and females in term, preterm at birth and preterm at term infant cohorts.

During the study period we obtained blood samples for telomere length analysis in 31 term born infants and 47 preterm infants (22 sampled within 48 hours of preterm birth and 25 sampled at term equivalent age). The characteristics of recruited term and preterm infants recorded at the time of sampling are displayed in Table 1. Within the term cohort, no baby was identified as having either blood culture positive sepsis or severe neonatal jaundice requiring neonatal exchange blood transfusion. In addition, the characteristics recorded at birth of preterm infants sampled at term equivalent age are shown in Table 2. As would be expected, birth gestation and birth weight of preterm infants (both preterm infants sampled at birth and those sampled at term) were significantly different (*p* = <0.01) when compared to term infants (Table 1). Birth gestation and birth weight in preterm infants sampled at birth (Table 1) and preterm infants sampled at term equivalent age (Table 2) were not significantly different (*p* = 0.28 and 0.18 respectively). There were no significant differences between the groups with respect to maternal age (Table 1). In each of the 3 cohorts, there were a greater number of male infants than female infants, however the proportion of each sex was similar in each group (Fig 1.) The Preterm infants sampled at term age demonstrated satisfactory postnatal growth in comparison to published UK data [62].

**Table 1:** Baseline characteristics at the time of sampling in term infants, preterm infants sampled at birth and preterm infants sampled at term equivalent age. Data are presented as mean (95% CI).

	Term (n = 31)	Preterm at term age (n = 25)	Preterm at birth (n = 22)
**Gestational age (weeks)**	39.85 (39.36–40.34)	38.11 (37.67–38.55)	27.99 (27.02–28.95)
**Weight (kg)**	3.58 (3.37–3.79)	2.27 (2.08–2.47)	1.14 (0.98–1.30)
**Maternal age (years)**	29.45 (26.80–32.11)	30.00 (27.76–32.24)	30.36 (27.56–33.17)

**Table 2:** Baseline characteristics for gestational age and weight at the time of birth in preterm infants that were sampled at term equivalent age (n = 25). Data are presented as mean (95% CI).

Baseline characteristics	Mean (95% CI)
**Gestational age at birth (weeks)**	27.30 (26.16–28.45)
**Time between birth and sampling (weeks)**	10.81 (9.56–12.06)
**Birth weight (kg)**	0.96 (0.84–1.09)
**Weight gain (g/kg/day between birth and sampling)**	19.6 (16.6–22.5)

### T/S Ratio

**Fig 2:**
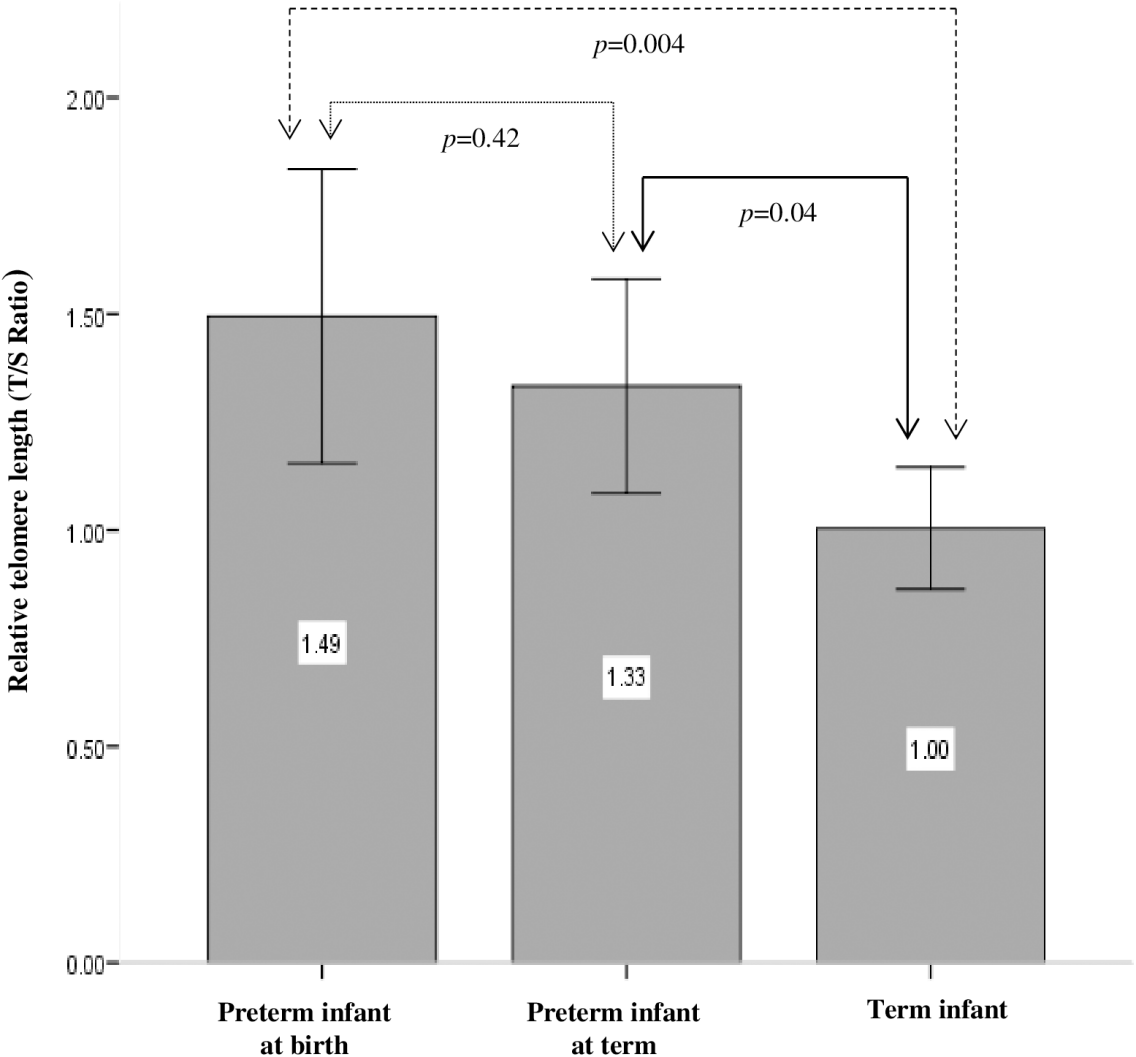
Relative telomere length (T/S ratio) in preterm infants at birth, preterm infants at term age and term born infants. Data are Mean (95% CI).

**Fig 3:**
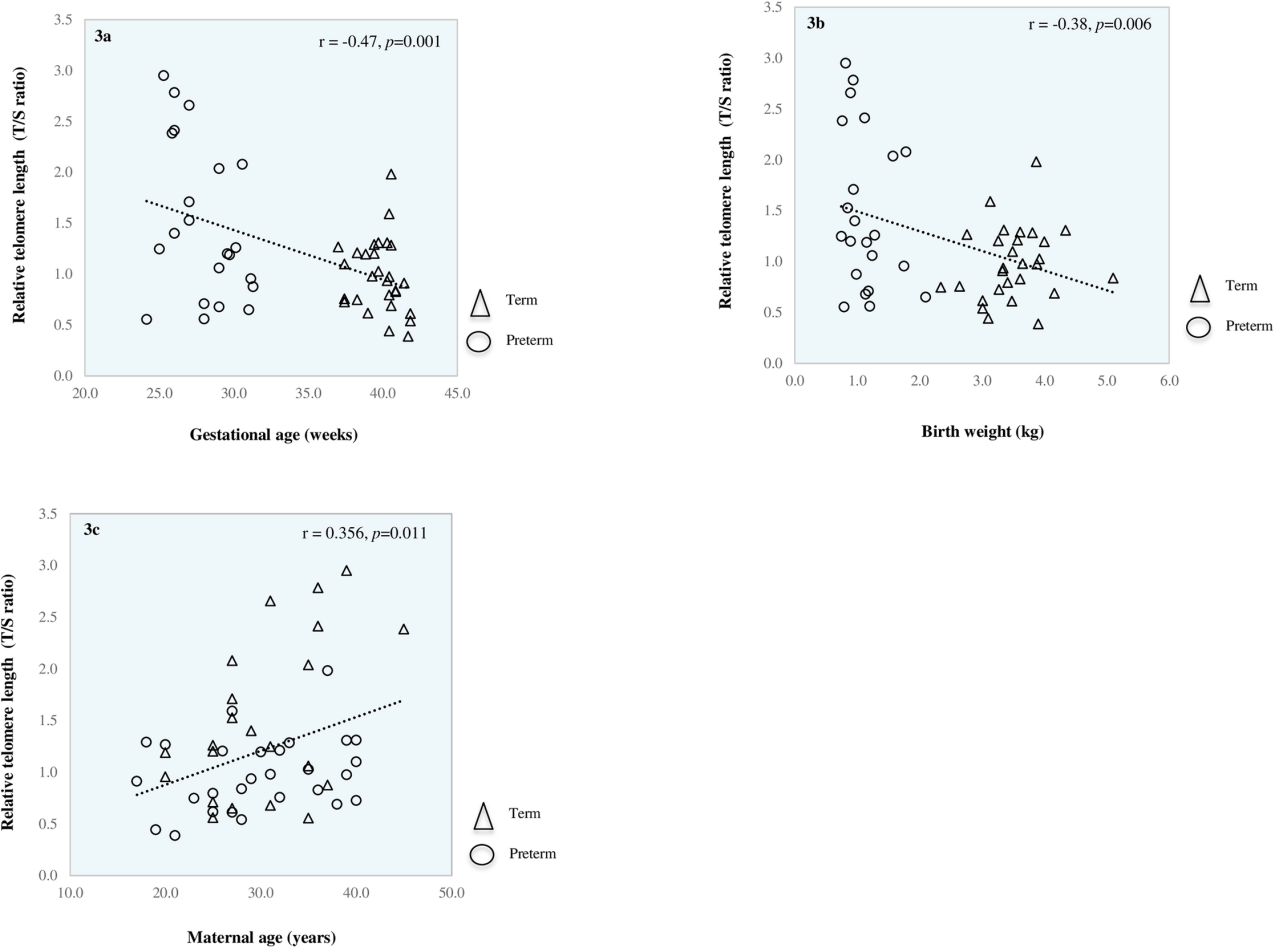
Relationship between relative telomere length (T/S ratio) and gestational age (a), birth weight (b) and maternal age (c).

Mean T/S ratio and T/S variability for preterm infants sampled at birth, preterm infants sampled at term and term born infants are shown in Fig 2. Mean T/S ratio and T/S variability of male and female infants overall and within each cohort are shown in Table 3. Finally, Fig 3A, 3B and 3C show correlation of T/S ratio with gestational age, birth weight and maternal age respectively in term infants and preterm infants sampled at birth.

**Table 3:** Gender distribution and mean (95% CI) T/S ratios of male and female infants included in the study overall and within each study cohort. *p* value signifies results from univariate ANOVA analyses of T/S ratios in males compared to females overall and within each cohort. (*Gender data missing in 2/31 term infants).

	Overall (n = 76)		Term (n = 29)*		Preterm at term (n = 25)		Preterm at birth (n = 22)	
	Mean (95% CI)	n	Mean (95% CI)	n	Mean (95% CI)	n	Mean (95% CI)	n
**Male**	1.29 (1.08–1.50)	49	1.13 (0.78–1.48)	18	1.25 (0.94–1.56)	17	1.22 (1.03–2.07)	14
**Female**	1.27 (1.05–1.48)	27	1.00 (0.68–1.30)	11	1.52 (1.03–2.00)	8	1.32 (0.98–1.82)	8
***p* value**	0.89		0.60		0.30		0.63	

We did not observe any difference in T/S ratios in males compared to female infants either overall, or within each cohort. However, our results show that both gestational age and birth weight were negatively correlated with T/S ratio (*p* = 0.001, *p* = 0.006 respectively) and that maternal age was positively correlated with T/S ratio (*p* = 0.011). Moreover, our data demonstrate a gradient effect with T/S ratios being highest in preterm infants sampled at birth and lowest in term infants, with term infant T/S ratios being significantly shorter than those of both preterm infants sampled at term (*p* = 0.04) and preterm infants sampled at birth (*p* = 0.004) (Fig 2). Cohen’s d analyses revealed a large effect size when comparing T/S ratios between term and preterm infants sampled at birth (d = -0.85, r = -0.39) and a medium effect size when comparing T/S ratios in term infants versus preterm infants sampled at term equivalent age (d = -0.71, r = -0.33). Though there was a reduction in mean T/S ratio between preterm infants sampled at birth and preterm infants sampled at term equivalent age, this was not statistically significant (*p* = 0.42).

For reasons highlighted in the discussion section, we were only able to obtain longitudinal telomere length samples (at the time of birth and at term equivalent age) in five preterm infants. A post hoc analysis of this longitudinal data enabled calculation of telomere attrition rate (Equation 2) between birth and term age.

$$\frac{T/S\;ratio\;(birth)-T/S\;ratio\;(term\;age)}{Number\;of\;weeks\;between\;birth\;sample\;and\;term\;age\;sample}$$

### Equation 2. Methodology for calculation of telomere attrition rate in five preterm infants sampled at birth and at term equivalent age

**Fig 4:**
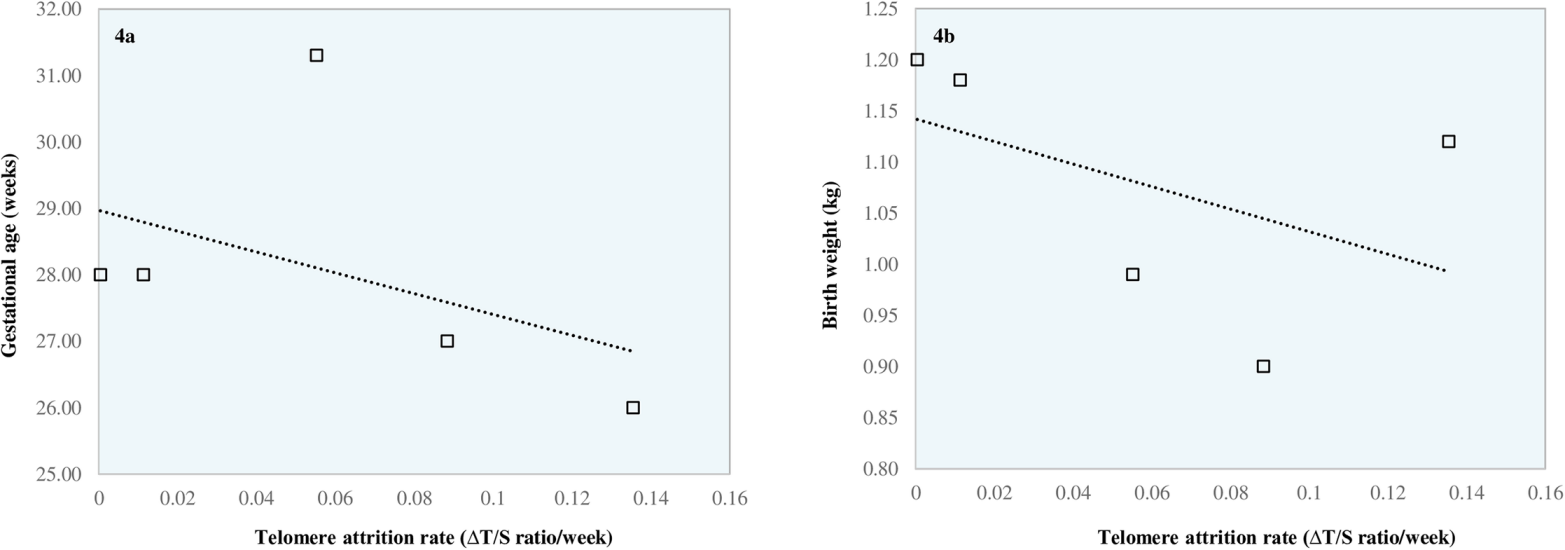
Relationship between telomere attrition rate (Δ T/S ratio per week) and birth gestation (a) and birth weight (b) in preterm infants with longitudinal samples.

Table 4 shows that in agreement with the cross sectional data described above, a reduction in T/S ratio was observed between the time of birth and term equivalent age in all five babies. However, the magnitude of this reduction was highly variable between these five babies. Paired t-test analysis revealed no difference between birth sample T/S ratio and term sample T/S ratio (*p* = 0.07) for these 5 babies (in keeping with cross sectional data presented in Fig 2. indicating no difference between birth sample T/S ratio and term sample T/S ratio in preterm infants). Intriguingly, our results also suggest an inverse relationship between telomere attrition rate and both birth weight (Pearson Correlation -0.47) and gestational age (Pearson Correlation -0.44). However, this was not of statistical significance (*p* = 0.42 and *p =* 0.46 respectively). These data are shown in Fig 4A and 4B.

**Table 4:** Longitudinal data and T/S ratios collected for five preterm infants. Data are Mean (95% CI).

Patient	1	2	3	4	5	Mean (95% CI)
**Gestation at Birth (weeks)**	27.00	26.00	31.30	28.00	28.00	28.06 (25.59–30.53)
**Birth weight (kg)**	0.90	1.12	0.99	1.20	1.18	1.08 (0.92–1.24)
**T/S ratio (Birth)**	2.66	2.41	0.87	0.56	0.71	1.44 (0.19–2.69)
**Gestation at term sample (weeks)**	37.00	37.43	38.00	36.86	36.86	37.23 (36.62–37.84)
**Weight (kg) at term sample**	1.93	3.06	1.95	3	2.72	2.53 (1.84–3.22)
**T/S ratio (term)**	1.77	0.86	0.50	0.56	0.59	0.86 (0.20–1.52)
**Number of weeks between sample 1 and sample 2**	10.00	11.43	6.71	8.86	8.86	9.17 (7.02–11.33)
**Change in T/S ratio between sample 1 and sample 2**	-0.884	-1.549	-0.37	-0.003	-0.123	-0.586 (-1.375–0.204)
**Telomere attrition rate/week (ΔT/S ratio/week)**	-0.0884	-0.1355	-0.0552	-0.0004	-0.0139	-0.0587 (-0.127–0.010)

## Discussion

In this study we assessed whole blood leukocyte telomere length in a cohort of preterm and term born infants shortly after birth, and for the first time, we also assessed telomere length in preterm infants at term equivalent age. Our data do not support our initial hypothesis that telomere length is significantly shortened by term equivalent age in preterm infants and in comparison to term infants. On the contrary, our results show that preterm infants at term equivalent age have significantly *longer* telomere lengths than term born infants. However, in keeping with other published data, our results indicate a significant decline in telomere length with advancing gestational age at birth [50–52]. We also observed a positive correlation between maternal age and T/S ratio, suggesting that older mothers deliver babies with longer relative telomere lengths. The strengths of this study are that it represents the only study to have directly compared telomere length measurement in preterm infants, assessed at term age and term born infants using a well established methodology. Potential limitations include the limited number of preterm infants in which we were able to obtain both birth and term age (longitudinal) telomere lengths and the observation that our ‘healthy’ comparator group of term infants cannot, by definition, be classified as entirely ‘healthy’ by the observation that they required blood testing. However, this latter point is merely reflective of the fact that obtaining research blood samples in completely healthy babies is not ethically sound.

Telomere length in preterm infants is a largely understudied area and to the best of our knowledge, only three other studies have assessed telomere length in preterm infants *in comparison to term born babies*: Friedrich *et al*. found no significant difference between the two groups when they assessed cord blood leukocyte telomere length [51], however Menon *et al*. and Ferrari *et al*. found significantly shortened leukocyte and placental telomere lengths respectively in term born infants compared to preterm infants born with intact membranes [50, 51, 53]. Interestingly, telomere length in preterm infants born following preterm pre-labour rupture of membranes was not different to term born infants and was also significantly shorter than preterm infants born with intact membranes. Furthermore, Ferrari *et al*. provided data to support the hypothesis that unexplained stillbirths are associated with placental telomere attrition by demonstrating a reduction in placental telomere length between stillbirths and term born infants [53]. Moreover, the placenta is known to contain sub-populations of karyotypically abnormal trophoblasts, which may have significant ramifications on telomere length measured in the Ferrari paper [63]. Indeed Ferrari *et al*. noted failed karyotype analysis in 15/42 stillborn cases.

While these findings (summarised in Table 5) offer interesting and novel insights into the physiological relevance of the events associated with telomere attrition that may lead to normal labour, preterm labour or unexplained stillbirth, none provide information on telomere length in the critical aberrant period of development that preterm born infants are exposed to in the neonatal intensive care unit during the weeks following preterm birth.

**Table 5:** A summary of studies to date that have assessed telomere length in preterm infants in comparison to term born infants or age matched fetus’.

Study	Friedrich *et al*	Holmes *et al*	Menon *et al*	Ferrari *et al*
**Group assessed**	Term (n = 11) versus preterm (n = 15)	Preterm (n = 5) versus age matched fetuses (n = 8)	Term (n = 35) versus preterm infants born with intact membrane (PTB, n = 69) and preterm infants born following preterm prelabour rupture of membranes (pPROM, n = 28)	Term (n = 43) versus PTB (n = 8), pPROM (n = 7) and stillborn (n = 42)
**Sample type**	Leukocytes from cord blood	Leukocyte from venous blood	Leukocytes from cord blood and DNA from placental tissue (n = 5 PTB, n = 5 pPROM, n = 8 term)	Placental tissue
**Methodology**	TRF analysis	TRF analysis	qRT-PCR	qRT-PCR
**Measure**	Absolute telomere length	Absolute telomere length	Absolute telomere length	Relative telomere length
**Key Findings**	No difference in telomere lengths of term (>37 weeks GA) versus preterm (<37 weeks GA). Significantly shorter telomeres in very low birth weight compared to low birth weight preterm infant. Significant decline in telomere length between 27 and 32 weeks gestation	Significant decline in telomere length in preterm infants sampled longitudinally between 23–35 weeks GA. No difference in telomere length observed in fetuses measured longitudinally over the same gestational period	Telomere length significantly longer in PTB (<37 weeks GA) compared to term born (>37 weeks GA) and pPROM (<37 weeks GA). No difference in telomere length between term and pPROM	Confirmation of findings by Menon *et al*. Significantly shorter telomere length in stillbirths (fetal death >22 weeks GA) compared to term (>37 weeks GA) and PTB (<37 weeks GA). No difference in telomere lengths of stillbirths compared to pPROM (<37 weeks GA)
**Reference**	45	46	44	47

Our own data contradict the findings of Friedrich and Menon and are fundamentally different to those presented by Ferrari, who examined placental telomere length. We propose that our finding that telomere length is longer in preterm infants sampled at birth compared to term born infants might be explained by a period of high cell turnover and replicative stress during a period of growth in the final weeks of pregnancy in the term infant that does not occur in those born preterm. As such, we suggest that the most prominent factors influencing telomere length in neonates are gestational maturity and birth weight (which are intrinsically linked except for where fetal growth restriction has occurred). Nonetheless, given the high variability in telomere lengths shown by our own and others’ data [26, 27, 48, 49, 64, 65], we suggest that there may also be other as yet undiscovered determinants of telomere length in newborns, which might include both genetic and epigenetic factors. Indeed other studies indicate heritability of telomere length and significant correlations between offspring telomere length and parental age [66–73]. Our own results and those of others indicate that this pattern is present at birth [64].

In light of previous observations by Holmes *et al*. who found a significant shortening of telomeres in the weeks following preterm birth [52], the finding that telomere length is not shortened at the time of term equivalent age in the preterm infant was unexpected. However, Holmes’ study only examined 5 preterm infants and thus may have been underpowered. We propose that there may be at least two possible explanations for this finding. Firstly, it is possible that the results from our study reflect a slow telomere attrition rate in the preterm infant during the initial weeks after birth, arising from slow replication and cell turnover. Indeed, many preterm infants undergo an initial phase of slow early growth (in comparison to fetal growth rates) [74, 75]. Alternatively, an opposing view arises from observations by others, who have noted that following birth, lymphocyte expansion rate occurs independently of gestational age [76–78]. In order to sustain rapid expansion of immature lymphocytes in the first few weeks of life, whilst evading telomere loss and subsequent entry into cell cycle arrest, it is possible that telomeres may be lengthened. In support of this, previous work has demonstrated up-regulation of telomerase and lengthened telomeres in response to stimulated expansion of naïve B lymphocytes isolated from adults and young children [79]. Should telomerase expression elicit a similar effect during the expansion of immature lymphocytes in the neonatal period, one might expect a greater population of cells with longer telomeres in the preterm group that were sampled some weeks after birth (at term equivalent age) in comparison to term born controls sampled within 48 hours of birth. Therefore it is also possible that our results can be explained by differences in the actions of telomere maintenance mechanisms between the study cohorts.

To further investigate these areas of uncertainty, it would have been beneficial to assess preterm infant telomere length longitudinally in all preterm babies recruited to our own study. This would additionally unmask any potential hidden effects attributable to a large spread in data as a result of variable genetic and epigenetic influences. Despite this being our original intention, obtaining samples for telomere length analysis at birth and at term equivalent age proved difficult as a number of preterm babies were either discharged from hospital or transferred back to their local neonatal unit prior to term age. From the five preterm infants in whom we were able to collect longitudinal data (Table 4), we demonstrated a reduction in telomere length between birth and term age. Intriguingly our data additionally suggests an inverse relationship between telomere attrition rate and both birth weight and gestational age (Fig 4A and 4B). Though we acknowledge that the sample size is small and there is no statistical significance, these preliminary data raise, for the first time, the biologically plausible hypothesis that telomere attrition rate in preterm infants maybe associated with the degree of prematurity, with the most preterm infants manifesting higher telomere attrition rates. Naturally, these data require confirmation in larger studies evaluating longitudinal telomere measurements in preterm infants. Our data also suggest that advanced maternal age is associated with increased relative telomere length in newborn infants. Okuda *et al* demonstrated a similar association, albeit using a telomere restriction fragment (TRF) methodology to measure newborn telomere length [64]. This finding is of interest and warrants replication in light of the increase in maternal age at time of first pregnancy that has been observed over recent years [80]. However, the relationship we describe may be a confounding variable given the relationship between maternal telomere length and newborn telomere length observed by others [81, 82]. The increase in vitro fertilisation methods over the past two decades may also be a relevant factor but in our own cohort, the majority of preterm births (93%) were as a result of a natural pregnancy.

It may be that analysis of average telomere length in the preterm infant at term equivalent age cannot act as a suitable marker of the aged phenotype observed. Perhaps rather than measuring telomere length at term equivalent age in preterm infants, it may be more prudent to measure them further downstream e.g. during later childhood or adolescence. This has proved to be informative in previous studies where shortened telomere length was shown to be associated with respiratory morbidity in the ex-preterm infant [34, 83]. However, the disadvantage of doing this would be the introduction of known and unknown confounding variables (i.e. lifestyle and epigenetic changes) which may influence telomere length [84]. Alternatively, there are a number of other putative markers of cellular senescence that may have more relevance to the detection of senescence associated with preterm birth that require further investigation. A plausible alternative candidate is SIRT 1, which is known to be down-regulated in association with insulin resistance, cardiovascular disease and metabolic disease. Moreover, SIRT 1 down-regulation is known to be associated with accelerated cord blood endothelial progenitor cell senescence in preterm infants [85]. Likewise, cell cycle regulators CDKN2A and CDK1A are known to be linked with ageing in adults and therefore warrant investigation in the newborn population [86–88].

In conclusion, our data indicate that preterm infants assessed at term equivalent age manifest longer telomere lengths than term born infants. In addition, our data and that of other groups [44, 64, 84, 89] show considerable variability in telomere length in preterm and term infants, suggesting that other mechanisms may exist alongside gestational maturity that are as yet unexplored determinants of telomere length. This high level of variability leads to a degree of overlap between the data in each of the cohorts assessed here and as such, we suggest that our data should be replicated by other groups and that future work in this area should evolve to examine a panel of markers of cellular senescence longitudinally. Furthermore, our findings lead us to speculate whether, as a response to preterm birth, there are mechanisms as yet not understood that serve to up regulate telomerase. These should be the focus of future research.

## Supporting information

S1 File (PATENT data file for PLoS One_170316.xls). Anonymised patient data collected for the study.
(XLSX)
